# Sphenoid Sinus Metastasis as the Presenting Manifestation of a Prostatic Adenocarcinoma: Case Report and Overview of the Literature

**DOI:** 10.1155/2012/819809

**Published:** 2012-11-03

**Authors:** I. Puche-Sanz, F. Vázquez-Alonso, J. F. Flores-Martín, H. Almonte-Fernández, J. M. Cózar-Olmo

**Affiliations:** Department of Urology, Hospital Universitario Virgen de las Nieves, Avenida Fuerzas Armadas 2, 18014 Granada, Spain

## Abstract

Although a metastatic presentation of an occult prostatic adenocarcinoma is not uncommon, the majority of these patients present with bone metastasis affecting the axial skeleton. Cranial metastases to the paranasal sinuses are extremely rare. A 56-year-old man presented with loss of vision and numbness of the right side of the face. Computed tomography (CT) scan and cranial magnetic resonance imaging (MRI) revealed a mass invading the sphenoid sinus. The patient underwent surgery to remove the lesion, and the histopathological examination suggested metastasis of an adenocarcinoma, with positive staining to prostatic specific antigen (PSA). However, serum PSA was 4 ng/mL, and the patient did not report any lower urinary tract symptoms or bone pain. Transrectal ultrasound-guided biopsy revealed prostatic adenocarcinomas with a Gleason score of 8 [4 + 4]. The subsequent treatment consisted of radiotherapy and androgen deprivation, followed by first- and second-line chemotherapy (docetaxel and cabazitaxel) when the disease progressed. The patient achieved a good response with the last cycle of cabazitaxel and after a 5-year followup is currently alive. Cranial metastases of prostate adenocarcinoma are rare, and there is currently no standard treatment for these patients. Whenever possible, surgery combined with radiotherapy and hormonotherapy is the recommended option.

## 1. Introduction

Metastatic tumors to the paranasal sinuses are rare. Kidney (renal cell carcinoma), lung, breast, testis (seminoma), gastrointestinal tract, and thyroid gland are, in order of frequency, the most common locations of the primary tumors that give origin to these metastases [1–3]. The sphenoid sinus is the most frequently involved, followed by the maxillary. In spite of the fact that a metastatic presentation of an occult prostatic carcinoma is not uncommon, the vast majority of these patients present with bone metastasis affecting the axial skeleton. Metastasis to the sphenoid sinus is an extremely rare event with less than 10 documented cases reported in the English literature [4]. We present an uncommon case of prostatic adenocarcinoma presenting with an extensive sphenoid sinus metastasis that, unlike the previous cases reported so far, has responded well to treatment and has achieved a long survival.

## 2. Case Report

**Figure 1:**
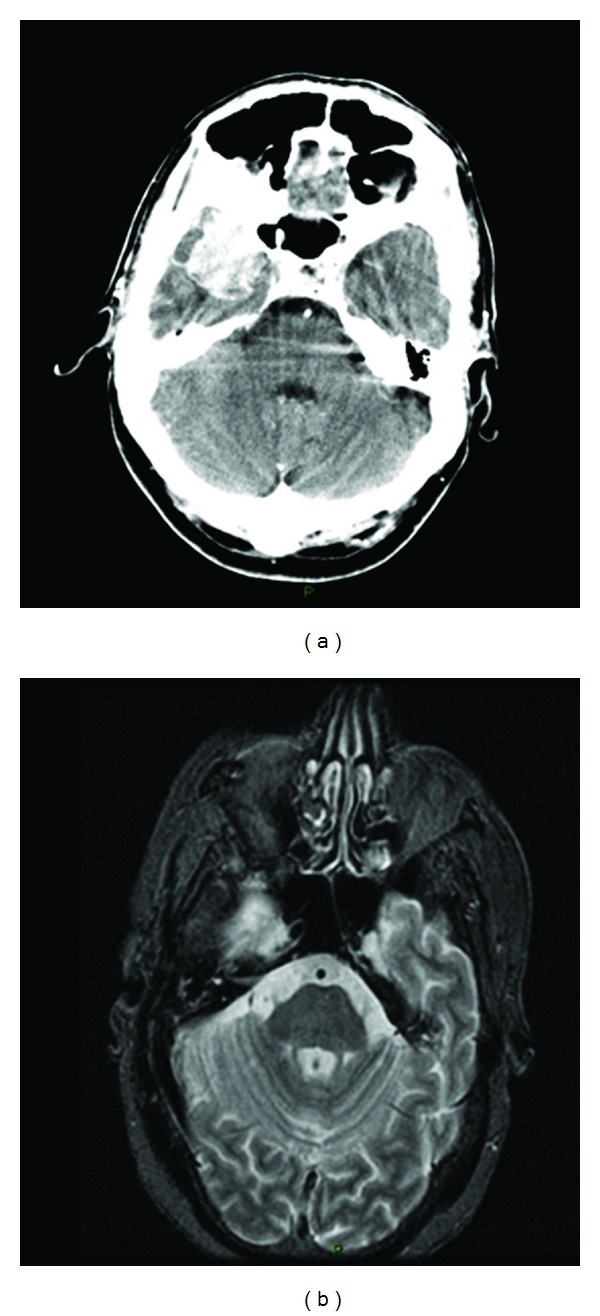
CT scan (a) and MRI (b) revealed a 4.5 × 4.5 × 3-cm mass that enhanced with contrast, in the right greater wing of the sphenoid bone which was invading the anterior pole of the temporal lobe and the sphenoid sinus.

**Figure 2:**
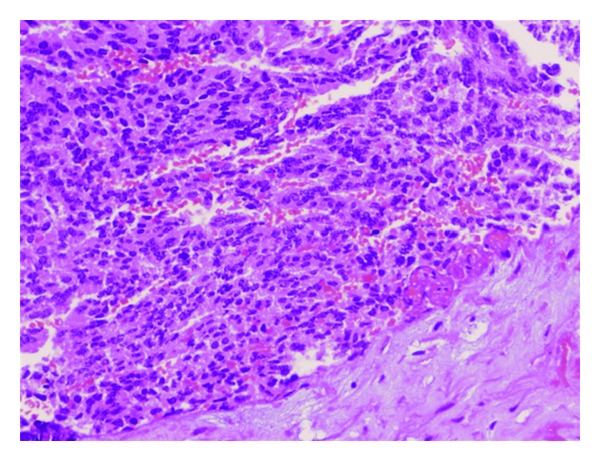
Hematoxylin-eosin (H-E) staining showing groups of glandular cells with different grades of atypia.

**Figure 3:**
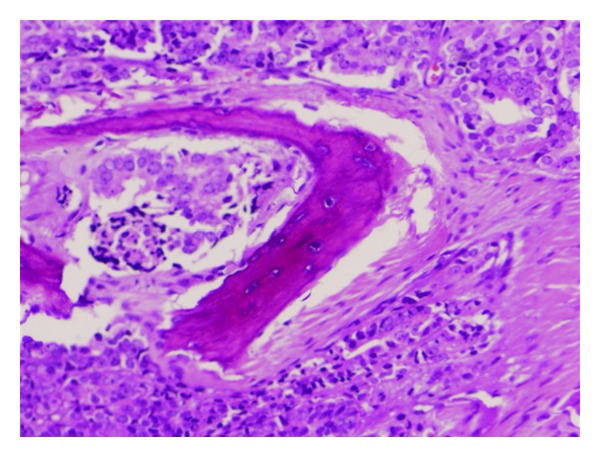
In this figure, the cellular invasion of the sinusal bone can be observed.

**Figure 4:**
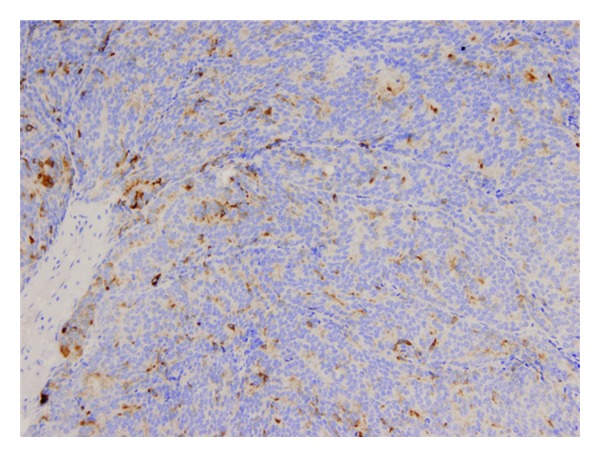
The cells showed focal and irregular positive staining for prostate specific antigen (PSA).

A 56-year-old male with no previous medical history of interest presented with a chief complaint of gradual right vision loss and numbness of the right side of the face. Computed tomography (CT) scan and cranial magnetic resonance imaging (MRI) revealed a 4.5 × 4.5 × 3 cm mass in the right greater wing of the sphenoid bone invading the anterior pole of the temporal lobe and the sphenoid sinus (Figure 1). A radical surgical approach was undertaken to remove the lesion. The histopathological study showed synaptophysin+, chromogranin+, PSA+, CK7−, CK20−, CD56−, TTF1−, CA19.9− and thyroglobulin−, and suggested metastasis of an adenocarcinoma (Figures 2, 3, and 4). Given the positivity for prostatic specific antigen (PSA), a transrectal ultrasound-guided biopsy was scheduled. The patient did not report any lower urinary tract syndrome or bone pain, and the serum PSA level was 4 ng/mL. However, on digital rectal examination the prostate had a stone-hard consistency, and the subsequent biopsy confirmed a prostatic adenocarcinomas with a Gleason score of 8 [4 + 4] in the right lobe. The bone gammagraphy was negative but the PET/CT-scan revealed a vertebral metastasis at C2 level. Treatment consisted of cranial and vertebral radiotherapy combined with LHRH analogues and corticosteroids. The patient showed a good response with rapid regression of the neurologic symptoms, PSA decrease, and elimination of the metastases. Two years later, PSA level raised and bicalutamide was added to the treatment, although one year later PSA raised again and bicalutamide was withdrawn. The following year, PSA raised again and a PET/CT scan revealed pelvic nodes involvement, so the patient was started on docetaxel-prednisone showing a stabilization of the disease. However, the PSA level kept on rising. Therefore, a year later, the patient continued on second-line cabacitaxel, showing a good response, with stabilization of the disease and PSA decrease. Five years after the diagnosis, the patient is still alive and has an acceptable quality of life, except for a slight ataxia and distal tremor, probably secondary to the treatment.

## 3. Discussion

Primary sinusal tumors account for approximately only 0.3% of all tumors [1]. Metastatic tumors to the paranasal sinuses are an exceptional event. Approximately, only 1% of the patients with prostate cancer will present any kind of manifestation in the head and/or neck [5]. The most common metastatic sites of the prostatic adenocarcinoma are the pelvic lymphatic nodes and the bones of the axial skeleton. Intracranial metastases are unusual, and when they occur, the diagnosis of prostate cancer is already made and the disease is already disseminated [6]. It is exceptional that a cranial metastasis appears as a first manifestation of a prostate cancer, as it is our case.

Some authors estimate that up to 10–20% of prostatic tumors are firstly diagnosed by their metastatic manifestations [6]. The way of distant dissemination of the metastases is either lymphatic or hematological. Hematological dissemination normally occurs throughout the intervertebral venous plexus of Batson. That fact would explain the most frequent involvement of the axial skeleton as a preferred metastatic site. Furthermore, it would also justify intracranial dissemination to the leptomeninges, which is the most common intracranial metastatic site. However, in the case of metastases to the orbit, they arrive necessarily from an arterial way [6], by means of tumoral emboli that overcome the pulmonary filter. 

The clinical presentation of the metastases in paranasal sinuses is similar to primary tumors in the same location. Some of the most frequent symptoms are headache, loss of vision, diplopia, facial numbness, loss of hearing, and other symptoms related to cranial pairs affection (II to VIII) [1, 3, 7]. Epixtasis is characteristically associated with metastases from renal cell carcinoma [8] and thyroid cancer [2]. Certain grade of suspicion should be maintained in case of persistent, antibiotic-resistant sinusitis and in case of the presence of risk factors for cancer in any location.

The CT scan and the MRI are essential for the diagnosis of metastases within the paranasal sinuses, as they reveal the size of the lesion and its extension to the adjacent structures, such as the orbit or the brain. There is not any specific radiological sign to differentiate metastases from a primary intracranial tumor. For instance, radiological features of a meningioma en plaque could be very similar to those of a retroorbital metastasis of a prostatic adenocarcinoma, as they both share an osteoblastic pattern. Therefore, histopathology study is crucial to reach a correct diagnosis, showing a sinusal mucosa with unspecific glandular architecture and positive to anti-PSA staining [9].

The elective treatment depends on the stage of the disease and the general condition of the patient. Due to the shortage of cases, there is no current standard of care for these patients. On the other hand, the early start of the treatment seems to produce a better control of the symptoms [6]. Radiotherapy alone or combined with androgen deprivation allows a rapid regression of the symptoms. In absence of consistent data, the combination of cranial surgery, radiotherapy and androgen deprivation seems to be the safer option to achieve a prolonged survival in those patients in which surgery is feasible and the disease is not widely disseminated [7]. Most authors agree that the role of surgery for the paranasal sinuses metastases should be exclusively limited to the diagnosis (biopsy) and to the palliation of the symptoms (mass reduction). A radical surgery approach in the treatment of the paranasal sinuses metastases could result in an incomplete, mutilating, and ineffective treatment, except in the case of easily approachable and unique metastasis [3]. However, in our case, the radical approach may have helped the long-term success.

The prognosis of metastatic prostatic disease to paranasal sinuses is not well documented, although it is generally considered to be unfavorable [10]. A review of periorbital prostatic metastases reported a survival of 16.3 months, showing no statistically significant differences when compared to other prostatic metastases [6]. However, there are cases with longer survival rates [7], as it is our case.

To conclude, we want to highlight that prostate cancer should always be considered within the differential diagnosis of any mass appearing in the cranial bones of aged patients with any neurological disorder, even if no urological symptoms are reported. Correct diagnosis is essential, as these patients may achieve prolonged survival with early treatment.
